# Acute vulvar pain in a lady with post circumcision inclusion cyst of the vulva containing stones: a case report

**DOI:** 10.1186/1472-6874-14-2

**Published:** 2014-01-06

**Authors:** Wondimu Gudu

**Affiliations:** 1 Department of Gynecology and Obstetrics, Karamara Regional Referral Hospital, Jijiga, Ethiopia

**Keywords:** Vulva stone, Epidermoid inclusion cyst, Female genital cutting

## Abstract

**Background:**

Despite global eradication efforts, female genital cutting is still deep routed practice in some parts of Asia and East Africa. The crude and unscientific natures of the practice lead to many complications. Epidermoid inclusion cysts of the vulva are one of the late complications of female genital cutting and typically present as painless cystic swellings. But clinical presentation as ‘stone’ containing, hard vulvar mass is reported only once in the literature and presentation with acute vulvar pain has never been documented.

**Case presentation:**

A 21 yrs old, Ethiopian, unmarried, lady presented with sever acute vulvar pain, discharge, and a stony hard vulvar swelling 13 years after type-III female genital cutting (infibulation). Surgical excision of the cyst, which contained two ‘stones’ inside it, and defibulation were done. Histopathology confirmed calcified, keratinizing epidermoid inclusion cyst of the vulva.

**Conclusions:**

Clinicians, in areas where female genital cutting is prevalent, should be aware of such unusual late vulvar complication of the practice which might be misdiagnosed for other solid vulvar swellings and be familiar with the appropriate management.

## Background

Female genital cutting is deep rooted practice in East Asia and north-east of Africa. It is associated with distressing immediate and late complications. Epidermoid inclusion cysts of the vulva are one of the commonest late complications of female genital cutting. These cysts occur as a result of the implantation of the epidermal keratinized squamous epithelial cells and sebaceous glands into the dermis in the line of circumcision scars and typically present as painless cystic vulvar swellings. Calcifications inside these cysts very rarely lead to formation of ‘vulvar stones’ which are the result of delayed presentation of patients. Extraction of the stones and complete excision of the cysts is the optimal management. We report, here, a case of stone containing post circumcision inclusion cyst of the vulva presenting with unusual acute vulvar pain in a lady who had type III female genital cutting during her childhood.

## Case presentation

A 21 yrs old single, nulligravid lady presented with excruciating vulvar pain and swelling of 3 days associated with itching and difficulty of walking. She had also thick discharge from the site of the swelling, dysuria and frequency.

The lady noticed a progressively increasing vulvar swelling since 4 years before her current presentation which initially was painless. But since a year back she had had recurrent episodes of sudden increases in size of the swelling with itching and pain which were subsiding after spontaneous ulceration followed by extrusion of thick ‘sand-like’ discharge from the swelling. She used to take non prescription antibiotics from pharmacies.

The lady underwent circumcision (infibulation) at the age of 8 years. Her menstrual cycles were unremarkable. She had no abdominal pain or vaginal discharge. She had no history of genital trauma or any similar swellings in other body parts. She had no known medical illnesses.

On physical examination she was acutely sick looking with difficulty of walking. Her vital signs were stable. The remarkable findings were on genital examination which revealed swollen & glistening vulva with scattered areas of dark tiny old scars on the skin. The labiae were completely fused having only a small (1 cm) opening just above the posterior fourchette. There was a palpable 4 cm by 3 cm sized, irregular, hard mass located in the midline along the fused labiae with a thick non offensive discharge extruding through pin pointed defect on the overlying skin at the cranial end.

**Figure 1:**
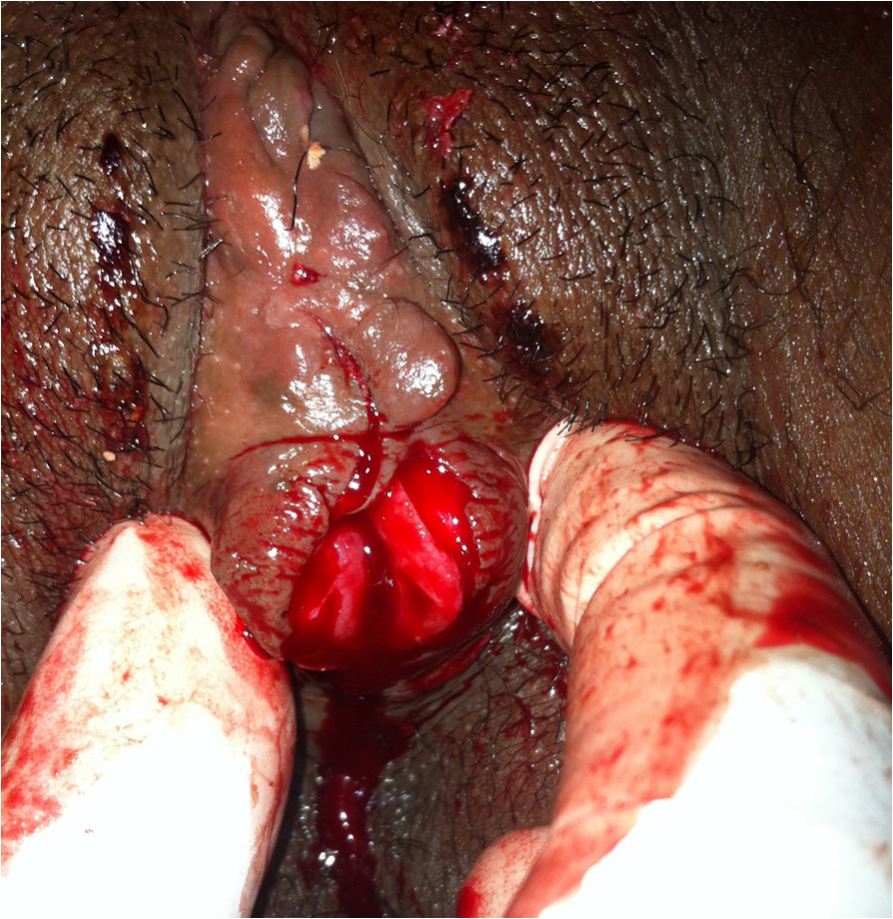
**The vulva after partial incision.** Picture taken after incision of the lower half of the fused labiae and extraction of the 1^st^ stone; thick discharge is visible through a dimple at the upper end of the fused labiae with underlying 2^nd^ stone before full incision.

**Figure 2:**
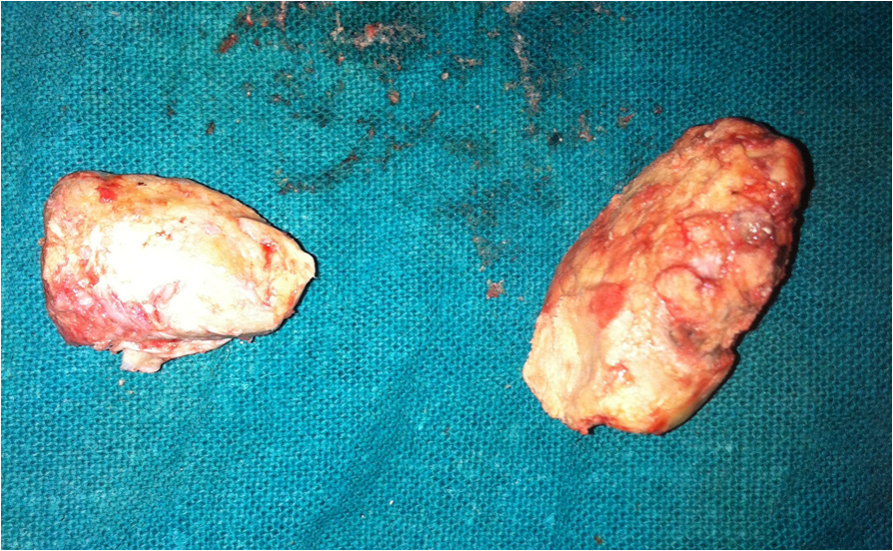
The two stones after extraction.

**Figure 3:**
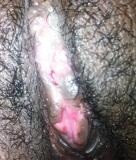
**The vulva on 7**^**th **^**post operative day.** Well separated (defibulated) & healed labial wound edges.

The laboratory tests were unremarkable except for pyuria on urinalysis. With a clinical diagnosis of super infected vulvar tumour, possibly infected post circumcision vulvar cyst, she was started on therapeutic cloxaciline and was counselled on surgical management with concurrent defibulation for which she consented. Under local (lidocaine) infiltration, incision was made on the mass which revealed thick walled biloculated cyst containing two discrete stones (Figures 1 and 2). Extraction of the stones, complete excision of the cyst wall and defibulation were done. The lady had marked improvement on her 7^th^ post operative day visit, with healed labial wound (Figure 3). The histopathology examination revealed a keratinized inclusion cyst of the vulva with no dysplasia. Chemical analysis of the stones was not done because of facility constraints.

## Discussion

Female genital cutting is gender based violence against women and a violation of basic human rights. Female genital cutting comprises all procedures involving partial or total removal of the external female genitalia or other injury to the female genital organs for non-medical reasons [1,2]. According to the World Health Organization, approximately 100 to 140 million girls and women worldwide have undergone some form of female genital cutting [1]. Estimated prevalence of female genital cutting in Africa ranges from 98% in Somalia to less than 1% in Uganda [1,2]. According to the 2005 Demographic and Health Survey (DHS) report, the prevalence of female genital cutting in Ethiopia was 74% with wide regional differences [3,4]. In the Somali region, where our case has been living, the prevalence was 97%; type-III (infibulation) being the commonest (60%) [4].

The World Health Organization has classified female genital cutting into four types: the first 3 including procedures of increasing invasiveness and the last one being a general category including unclassified genital injuries [1]. Type- III female genital cutting (infibulation) involves narrowing of the vaginal opening through the creation of a covering seal by cutting and sewing the labia minora and/or the labia majora, with or without removal of the clitoris [2]. This was evident in our case.

Immediate complications of female genital cutting include: severe pain, hemorrhage, infection, urine retention, and injury to nearby genital tissue. The long-term consequences include recurrent urinary tract infections, infertility, psychosexual disturbance, an increased risk of childbirth complications and epidermal inclusion cysts [5,6].

Epidermoid cysts are intradermal keratinizing cysts lined by squamous epithelium. The cysts result from ingrowths or implantation of epidermal tissues underneath other tissues. The accumulation of epidermal desquamations, secretions and other debris in a closed space leads to the formation of a cystic and often painless swelling that gradually increases in size over time [7]. The common sites of involvement are face, trunk, neck, extremities, and scalp. The vulva is a rare site [7].

Though histopathologically similar, the pathogenesis of epidermoid cysts of the vulva differ from that of cysts arising from hairy (acne prone) areas such as the face, neck which arise from the damage to the pilosebaceous unit [7,8]. But On the vulva, these cysts result from the burial of fragments of the skin following female genital cutting, vulval trauma, perineal tears and episiotomy [7,9]. Our case underwent infibulation in which the labial wound edges are usually closed with thorns or sutures by circumcisers lacking surgical skills which makes the incorporation of the keratinized epithelium and sebaceous glands into the dermis highly likely [8,9].

The frequency and delay from circumcision to development of inclusion cyst are extremely variable. The delayed development of inclusion cysts after circumcision is postulated to be secondary to unopposed estrogenic stimulation of the embedded epidermal tissue and sebaceous glands during anovulatory menstrual cycles of adolescence [8]. This was also evident in our case who noticed the vulvar cyst initially at the age of 17, nine years after circumcision.

The clinical presentation of patients is variable [7]. Most Epidermoid cysts are often asymptomatic. Pain, discomfort, or rupture of cysts during sexual intercourse or vaginal delivery may force patients to seek medical care early [9,10]. In spite of the presence of mild symptoms, our case neglected the cyst for many years which resulted in calcifications. The recurrent ‘sandy like’ discharges in the patient were possibly the result of pressure necrosis of the epidermis caused by the underlying stones leading to micro perforations which were healing spontaneously. This was evident by the tiny scattered dark scars on the skin overlying the cyst.

Complication of epidermoid inclusion cysts include rupture & release of keratin leading to intense inflammatory reaction, infection, hematoma and rarely carcinomas [9,11]. The fact that our case presented with an acute sever vulvar pain with a discharge through the pinpointed opening on the cyst surface, might suggest a possible manifestation of sever foreign body inflammatory reaction which forced her to seek emergency medical care unlike the previous recurrent episodes which were mild.

Diagnosis of epidermoid inclusion cysts is confirmed by Histologic examination which was done also in our case and revealed the typical finding of cyst lined by keratinized stratified squamous epithelium [7].

The Management of inclusion cysts is surgical excision which was done in our patient with concurrent correction of the infibulation (defibulation), which resulted in satisfactory clinical and cosmetic outcomes.

## Conclusions

Post circumcision epidermoid inclusion cyst of the vulva containing stones and presenting with acute vulvar pain is extremely rare. Health professional in areas where female genital cutting is prevalent, should be aware of such unusual late vulvar complications of the practice, which might be misdiagnosed for other solid vulvar dermatologic tumours and be familiar with the appropriate management.

## Consent

Verbal explanation was given to the case (the participant) and an informed written consent was obtained both for the writing up and taking the pictures. I assure that I will produce the consent form at any time upon request.

## Competing interests

The author does not have any competing interests with respect to the research, authorship and publication of this article.

## Authors’ contributions

All the activities related to the case report were done by the author.

## Authors’ information

WJG is an Obstetrician and Gynecologist at Departement of Obstetrics and Gynecology, Karamara regional referral hospital, Somali regional state, Jijiga, Ethiopia.

## Pre-publication history

The pre-publication history for this paper can be accessed here:

http://www.biomedcentral.com/1472-6874/14/2/prepub
